# Comparing the Health Effects of Ambient Particulate Matter Estimated Using Ground-Based versus Remote Sensing Exposure Estimates

**DOI:** 10.1289/EHP575

**Published:** 2016-09-09

**Authors:** Michael Jerrett, Michelle C. Turner, Bernardo S. Beckerman, C. Arden Pope, Aaron van Donkelaar, Randall V. Martin, Marc Serre, Dan Crouse, Susan M. Gapstur, Daniel Krewski, W. Ryan Diver, Patricia F. Coogan, George D. Thurston, Richard T. Burnett

**Affiliations:** 1 Department of Environmental Health Sciences, Fielding School of Public Health, University of California, Los Angeles, Los Angeles, California, USA; 2 McLaughlin Centre for Population Health Risk Assessment, University of Ottawa, Ottawa, Ontario, Canada; 3 ISGlobal, Centre for Research in Environmental Epidemiology (CREAL), Barcelona, Spain; 4 Universitat Pompeu Fabra (UPF), Barcelona, Spain; 5 CIBER Epidemiologia y Salud Publica (CIBERESP), Madrid, Spain; 6 Division of Environmental Health Sciences, Public Health Department, University of California, Berkeley, Berkeley, California, USA; 7 Department of Economics, Brigham Young University, Provo, Utah, USA; 8 Department of Physics and Atmospheric Science, Dalhousie University, Halifax, Nova Scotia, Canada; 9 Department of Environmental Sciences and Engineering, School of Public Health, University of North Carolina at Chapel Hill, Chapel Hill, North Carolina, USA; 10 Department of Sociology, New Brunswick Institute of Research, Data and Training, University of New Brunswick, Fredericton, New Brunswick, Canada; 11 Epidemiology Research Program, American Cancer Society, Atlanta, Georgia, USA; 12 Department of Epidemiology and Community Medicine, University of Ottawa, Ottawa, Ontario, Canada; 13 Slone Epidemiology Center, Boston University, Boston, Massachusetts, USA; 14 New York University School of Medicine, Tuxedo, New York, USA; 15 Population Studies Division, Health Canada, Ottawa, Ontario, Canada

## Abstract

**Background:**

Remote sensing (RS) is increasingly used for exposure assessment in epidemiological and burden of disease studies, including those investigating whether chronic exposure to ambient fine particulate matter (PM_2.5_) is associated with mortality.

**Objectives:**

We compared relative risk estimates of mortality from diseases of the circulatory system for PM_2.5_ modeled from RS with that for PM_2.5_ modeled using ground-level information.

**Methods:**

We geocoded the baseline residence of 668,629 American Cancer Society Cancer Prevention Study II (CPS-II) cohort participants followed from 1982 to 2004 and assigned PM_2.5_ levels to all participants using seven different exposure models. Most of the exposure models were averaged for the years 2002–2004, and one RS estimate was for a longer, contemporaneous period. We used Cox proportional hazards regression to estimate relative risks (RRs) for the association of PM_2.5_ with circulatory mortality and ischemic heart disease.

**Results:**

Estimates of mortality risk differed among exposure models. The smallest relative risk was observed for the RS estimates that excluded ground-based monitors for circulatory deaths [RR = 1.02, 95% confidence interval (CI): 1.00, 1.04 per 10 μg/m^3^ increment in PM_2.5_]. The largest relative risk was observed for the land-use regression model that included traffic information (RR = 1.14, 95% CI: 1.11, 1.17 per 10 μg/m^3^ increment in PM_2.5_).

**Conclusions:**

We found significant associations between PM_2.5_ and mortality in every model; however, relative risks estimated from exposure models using ground-based information were generally larger than those estimated using RS alone.

**Citation:**

Jerrett M, Turner MC, Beckerman BS, Pope CA III, van Donkelaar A, Martin RV, Serre M, Crouse D, Gapstur SM, Krewski D, Diver WR, Coogan PF, Thurston GD, Burnett RT. 2017. Comparing the health effects of ambient particulate matter estimated using ground-based versus remote sensing exposure estimates. Environ Health Perspect 125:552–559; http://dx.doi.org/10.1289/EHP575

## Introduction

Remote sensing (RS) and atmospheric chemistry models play an increasingly important role in exposure assessment for epidemiological and burden-of-disease studies. A wide array of products produced by several U.S. federal agencies, such as the National Aeronautics and Space Administration (NASA) and the U.S. Environmental Protection Agency (EPA), are now available. Sometimes, these models form the basis for more complex estimates combining ground-based data or several remote-sensing products.

Several recent epidemiological investigations have used remote sensing for the exposure assessment or as input into other health impact assessment or variable-imputation models. By combining retrievals of aerosol optical depth (AOD) from the Moderate Resolution Imaging SpectroRadiometer (MODIS) and Multi-angle Imaging SpectroRadiometer (MISR) instruments onboard the Terra satellite with the GEOS-Chem model, van Donkelaar et al. (2010) developed 6-year mean global estimates of PM_2.5_ at ~10 km resolution (van Donkelaar et al. 2010). These RS products were designed to avoid reliance on PM_2.5_ monitors because these RS products can offer information about PM_2.5_ in regions where PM_2.5_ monitors are not generally available or where there are concerns about PM_2.5_ data quality, as, for example, with Tapered Element Oscillating Microbalances (TEOMs). Researchers in Canada have used the van Donkelaar et al. (2010) estimates to assess the health effects of air pollution. Specifically, these PM_2.5_ estimates were significantly associated with incidence of diabetes (Chen et al. 2013) and diabetes mortality (Brook et al. 2013) and cardiovascular mortality (Crouse et al. 2012, 2015). These RS estimates have also been used to estimate the global mortality associated with PM_2.5_ (Evans et al. 2013; Lim et al. 2012).

A few studies have attempted to systematically compare the exposure estimates from ground-based versus RS models. Lee et al. (2012) developed national-level models using data from more than 1,300 ground monitors for PM_2.5_ (Lee et al. 2012). Their results indicated that within ~98 km of a monitor, the ground-based estimates predicted PM_2.5_ concentrations more accurately than the RS estimates discussed above (van Donkelaar et al. 2010). Beyond 98 km, however, the RS estimates were better predictors of ground-level PM_2.5_. For the most part, the estimates were highly correlated with each other, and the authors concluded that the differences in prediction capacity were fairly small. Another study compared NASA AOD retrievals to ground-based estimates derived from a generalized linear model that included ground information on land-use predictors and several statistical smoothing functions. The study concluded that the RS estimates were not generally better predictors than the ground-based models, and after applying smoothing functions in the models, there was little marginal benefit to the RS information on predicting ground-level PM_2.5_ (Paciorek and Liu 2009). Subsequent studies have found that ground-based observations can be better predicted using exposure models with RS estimates than without (Beckerman et al. 2013a, 2013b; Kloog et al. 2012b; Ma et al. 2014; Vienneau et al. 2013).

RS estimates of air pollution generally lack the fine-scale resolution (< 1 km) needed for use in environmental epidemiological studies that aim to understand small-area variations in exposure. To achieve horizontal downscaling of the RS estimates, hybrid approaches that combine variants of land-use regression models, which predict pollutant concentrations from land use such as road length, traffic density, or open space with RS measurements are being employed (Beckerman et al. 2013a, 2013b; Kloog et al. 2012b; Ma et al. 2014; Vienneau et al. 2013). Through statistical modeling, proxy information about likely locations of pollution at smaller spatial resolution than AOD pixels can essentially distribute the PM_2.5_ estimated from the AOD to its most likely locations within its pixel. These hybrid exposure estimates have been used in a number of epidemiological studies (Jerrett et al. 2013; Kloog et al. 2012a, 2013; Madrigano et al. 2013).

Although now in broader use, little is known about the impact of using RS estimates on predicted health effects as compared to either monitored data or hybrid models. In the present study, we used the American Cancer Society Cancer Prevention Study II (CPS-II) (Jerrett et al. 2005, 2009, 2013)—a well-documented, U.S.-wide prospective cohort study—to compare various RS, geostatistical, and hybrid models in the estimation of circulatory and ischemic heart disease (IHD) mortality associated with ambient PM_2.5_. Recently, several papers have been published using 1-km estimates of PM_2.5_ for the United States; some of these more spatially fine-grained estimates used ground data extensively (see, e.g., Lee et al. 2016; van Donkelaar et al. 2015b). Calibration with ground data likely improves the performance in the United States, where there is a large and spatially wide coverage of ground-based monitors. In other regions that lack extensive monitoring support, such calibration is more challenging. In this paper, therefore, we have included estimates that incorporate ground data and those that rely solely on RS retrievals. Including both allows us to directly assess the importance of ground data calibration.

## Methods

This section outlines the health data, exposure models, and statistical analyses performed in the present study. Further details are provided in “Methods. Detailed Description of the Individual and Ecological Variables included in the Cox proportional Hazards Models” in the Supplemental Material.

### Health and Demographic Data

In September 1982 and February 1983, volunteers enrolled participants in the CPS-II cohort. In total, 1,184,587 participants ≥ 30 years of age were enrolled at baseline. The participants were mostly friends and family members of the volunteers. Participants were recruited from all 50 states, Washington, DC, and Puerto Rico. They completed a four-page, self-administered survey with items on demographic, lifestyle, medical, and other variables, including residential address at baseline. The CPS-II was approved by the Institutional Review Board of Emory University, and participants provided informed consent prior to participation. Approval for the analysis in this paper specifically was obtained from the Ottawa Hospital Research Ethics Board and the Committee on the Protection of Human Subjects, University of California, Berkeley.

We geocoded participant residences at baseline, which were then used to assign several exposures at either the individual participant residence or census tract (CT) of residence (see Pope et al. 2015 for details). After making exclusions for missing residence information and for key covariates such as smoking, 668,629 participants remained in the analytical cohort used in this analysis. See Table S1 for a comparison of those included versus those excluded and for some commentary on the minor differences between the two groups.

Vital status from 1982 through to 2004 was ascertained using methods documented elsewhere (Jerrett et al. 2009). Briefly, in 1984, 1986, and 1988, vital status was determined by the study volunteers, with subsequent confirmation obtained by the corresponding death certificate. For deaths after 1989, computerized linkage to the National Death Index was used for follow-up (Calle and Terrell 1993). We focused on mortality from diseases of the circulatory system [*International Classification of Diseases, Ninth Revision* (ICD9) codes 390–459; *International Statistical Classification of Diseases and Related Health Problems* (10th Revision) (ICD10) codes I00–I99] for comparability with existing studies (see, e.g., Hoek et al. 2013). We also examined effects on IHD (ICD 9 codes 410–414; ICD10 codes I20–I25) deaths because this outcome had the largest effect sizes in the ACS cohort (Turner et al. 2016) and thus is amenable to assessing inter-model differences in the exposure assessment. Given evidence that long-term PM_2.5_ exposures may also be associated with diabetic deaths (Brook et al. 2013; Pope et al. 2015), we also examined effects on diabetic deaths (ICD10 code E11) as a supplementary analysis, although there were considerably fewer deaths attributed to this cause.

### Exposure Models

The models are summarized in Table 1 in terms of their spatial and temporal resolution, the types of data used to derive the estimates, and the cross-validation results. First, the RS data set, mentioned earlier, was developed with the MODIS and MISR satellites with scaling to ground level achieved via a chemical transport model (GEOS-Chem). These initial estimates were produced globally on a 0.1° × 0.1° (~9.8 km) for the years 2001–2006 (van Donkelaar et al. 2010). Additional RS estimates representing 2002–2004 were also included (van Donkelaar et al. 2015b). These updated estimates were provided at 0.01° × 0.01° (~1 km) resolution produced with an optimal estimation algorithm developed for MODIS observations and with the subsequent inclusion of ground-based observations through a globally applicable (van Donkelaar et al. 2016) geographically weighted regression that restricted monitors for training to > 100 km away. Scaling to years before 2004 followed the method of van Donkelaar et al. (2015a), which relied upon trend information (Boys et al. 2014) from the Sea-Viewing Wide Field-of-View Sensor (SeaWiFS) (Hsu et al. 2013) and MISR satellite instruments. Second, we assigned the Hierarchical Bayesian Model (HBMCMAQ) developed by the U.S. Environmental Protection Agency (EPA) (McMillan et al. 2010). This model fuses daily estimates from the Community Multi-Scale Air Quality (CMAQ) model with ground observations in a Bayesian modeling regime that essentially upweights the influence of the CMAQ predictions as a function of distance away from the monitor. These estimates were derived nationally for a ~36 km × 36 km grid. We averaged the daily estimates to a 3-year average of 2002–2004 (cf. Turner et al. 2016). Third, we assigned a Bayesian Maximum Entropy (BME) spatiotemporal geostatistical kriging model based on ground observations (~9.8 km) (Lee et al. 2012). This model was fit based on 1,364 *in situ* monitors. Fourth, we assigned a hybrid land-use regression model using only ground-based inputs where the first stage of the model was fit with a deterministic regression model with monthly pollution as the dependent variable and land use and traffic information as predictors, with the second stage consisting of a BME kriging model of the residuals (BMELUR). Predictions from the two models were combined post hoc to derive the exposure surface, which was averaged over the period 2002–2004. Finally, we developed the fifth model using a similar kriging–LUR approach that combined ground-based information with the RS estimates (BMELURRS) (Beckerman et al. 2013b). The variables in the final two models were selected with a deletion/substitution/addition algorithm, which relies on v-fold cross-validation to avoid over-fitting to the measured data.

**Table 1:** Model descriptions including spatial and temporal dimensions, auxiliary data, and cross-validation summary.

Model name	Model type	Spatial scale	Temporal scale	Ground data used	Other auxiliary data	Cross-validation methods and results
PM_2.5_ HBMCMAQ 02-04	Atmospheric chemistry with statistical data fusion	36 km × 36 km grid	2002–2004	Yes, used in the data fusion	Yes, meteorological data	Conducted for sub-area of the Northeastern and Midwestern parts of the continent with a 12 km × 12 km grid; 44 Federal Reference Method sites used for cross-validation results were found to track monitoring data temporal patterns well, but with some seasonal bias. Results outperformed exponential kriging model for bias and were slightly worse for Mean Square Error (MSE) (McMillan et al. 2010)
PM_2.5_ BME 02-04	Bayesian Maximum Entropy Space-time kriging	Predicted at centroids of 0.1° × 0.1° ~ 9.8 km × 9.8 km grid used for estimating the PM_2.5_ RS 01-06 (see below)	2002–2004	Yes, based on 1,318 monitors with monthly averages	No	Extensive cross-validation based on 146 leave-out sites with MSE generally less 5 for distances < 98.7 km from the cross-validation site. This model predicted ground-level concentrations more accurately than the PM_2.5_ RS 01-06, whereas at greater distances, the PM_2.5_ RS 01-06 outperformed the kriging model in terms of MSE (see below) (Lee et al. 2012)
PM_2.5_ BMELUR 02-04	Land use regression (LUR) model with Bayesian Maximum Entropy kriging of residuals from the LUR model	30 m × 30 m estimate centered on the target receptor location	2002–2004	Yes, based on 1,318 monitors with monthly averages	Yes, traffic density within 1 km of the monitor and open land, acres within 100 m	Model variables selected with a machine-learning algorithm that used v-fold cross-validation in the model selection. Approximately 10% of the data were held out for cross-validation (i.e., 146 ground sites). Cross-validation *r*^2^ ~ 0.8 with little evidence of bias or heteroskedasticity (Beckerman et al. 2013b)
PM_2.5_ BMELURRS 02-04	Land-use regression model with Bayesian Maximum Entropy kriging of residuals from the LUR model	30 m × 30 m estimate centered on the target receptor location	2002–2004	Yes, based on 1,318 monitors with monthly averages	Yes, remote sensing estimate at ~ 9.8 km and open land, acres within 400 m	Model underwent the same cross-validation as the PM_2.5_ BMELUR 02-04 above. Cross-validation *r*^2^ ~ 0.8 with no apparent sign of bias or heteroskedasticity (Beckerman et al. 2013b)
PM_2.5_ GWR RS 02-04	Based on aerosol optical depth from the MODIS satellite instrument scaled to prior years using AOD from the MISR and SeaWiFS satellite instruments and adjusted with geographically weighted regression	0.01° × 0.01° ~ 1 km × 1 km grid	2002–2004	Yes, used in the geographically weighted regression	Atmospheric Chemical Transport Model (GEOS-Chem); scaling to years before 2004 follows van Donkelaar et al. (2015a)	*r*^2^ = 0.79 (cross-validated); Uncertainty = *N* (–0.38, 1.49) μg/m^3^; RMSD = 1.5 μg/m^3^; based on comparison with 2004–2008 PM_2.5_ observed at 1,440 ground monitors (van Donkelaar et al. 2015b)
PM_2.5_ No GWR RS 02‑04	Based on aerosol optical depth from the MODIS satellite instrument scaled to prior years using AOD from the MISR and SeaWiFS satellite instruments	0.01° × 0.01° ~ 1 km × 1 km grid	2002–2004	No	Atmospheric Chemical Transport Model (GEOS-Chem); scaling to years before 2004 follows van Donkelaar et al. (2015a)	*r*^2^ = 0.62; Uncertainty = *N *(–0.87, 2.42) μg/m^3^; RMSD = 2.65 μg/m^3^; based on comparison with 2004–2008 PM_2.5_ observed at 1,440 ground monitors (van Donkelaar et al. 2015b)
PM_2.5_ RS 01-06	Based on aerosol optical depth from the MODIS and MISR satellite instruments	0.1° × 0.1° ~ 9.8 km × 9.8 km grid	2001–2006	No	Atmospheric Chemical Transport Model (GEOS-Chem)	*r*^2^ = 0.49 (noncoincident); *r*^2^ = 0.59; 1 – σ error = 1 μg/m^3^ + 15%; y = 1.07x – 1.75 (coincident) based on comparison with 2001–2006 PM_2.5_ observed at 1,057 ground monitors (van Donkelaar et al. 2010)
Notes: MISR, Multi-angle Imaging SpectroRadiometer; MODIS, Moderate Resolution Imaging SpectroRadiometer.						

### Statistical Models

We employed Cox proportional hazards regression to examine associations of PM_2.5_ exposure with death from diseases of the circulatory system and from IHD while controlling for likely individual and ecological confounders.

We used follow-up time in days from enrollment as the time axis. As in previous analyses (Jerrett et al. 2013), we stratified models by 1-year age categories, sex, and race (white, black, other). This stratification allowed each category to have its own baseline hazard. We included a comprehensive set of individual risk factor variables operationalized in a similar way to those used in previous studies of the CPS-II cohort (Jerrett et al. 2009; Krewski et al. 2009). We used ecological variables extracted from the 1990 and 2000 U.S. Census (U.S. Department of Commerce, Bureau of the Census 1993, 2004) in the ZIP code neighborhoods of residence to control for potential “contextual” neighborhood effects. We provide details in “Methods. Detailed Description of the Individual and Ecological Variables included in the Cox proportional Hazards Models” in the Supplemental Material.

As a sensitivity analysis, we estimated selected adjusted hazard ratios using multi-level models that included a random effect term for the county of residence (Jerrett et al. 2009). We also included variables controlling for the size of the metropolitan area of residence, which has been found to influence air pollution–mortality associations (Crouse et al. 2012; Jerrett et al. 2013), and for elevation because higher elevations have been related to cardiovascular mortality in this cohort and are generally associated with lower pollution levels (Krewski et al. 2000).

To assess overall model fit, we used the Akaike Information Criterion (AIC). We hypothesized that models with a better overall prediction had lower measurement error and would therefore have lower AIC values and larger coefficients in the Cox regression model.

Our assessments of model fit and effect size might be suggestive about which model provides the best prediction of mortality, but in observational studies, we have no way of knowing which model best reflects the true relationship between air pollution and survival. For our main results, therefore, we developed ensemble estimates that pooled the effects from every model into a single estimate. This method derived a weighted average of the coefficients from the various models with the weights defined in terms of the change in the AIC from that of the model with the minimum AIC (Buckland et al. 1997; Faes et al. 2007). Specifically, we computed the weights as follows:


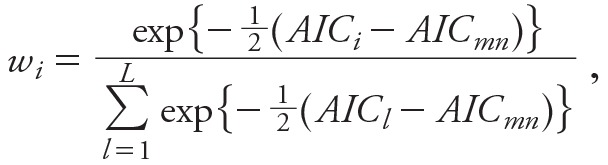
,

where *AIC_i_* is the Akaike Information Criterion of the *i*th model and *AIC_mn_* denotes the minimum Akaike Information Criterion among the *L* models examined. Because the number of parameters is identical in all models and because *AIC* = –2ln*lik* + 2*k*, where ln*lik* denotes the logarithm of the likelihood function and *k* denotes the number of parameters in the model, the ensemble weights *w_i_* can be written as


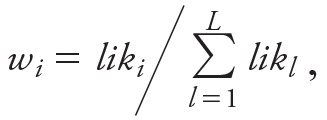
,

where *lik_i_* is the likelihood function for the *i*th model. In this case, we interpret the ensemble weights as a function of the likelihood and not necessarily of the *AIC*. However, in practice, we used the definition of the ensemble weights in terms of the *AIC* because the value of the likelihood for a study as large as the CPS-II cohort is too great to be calculated with standard computing software.

## Results

Descriptive statistics of the analytic cohort are shown in Table S1, which presents the average PM_2.5_ exposures across the strata of the covariates. Few variables appear to be associated with PM_2.5_. Black participants tended to have higher exposures, although they account for a very small proportion of the cohort (3.8%). A slight inverse trend exists in the relationship between pollution and education (i.e., those with higher education have generally lower pollution levels).

Table 2 presents descriptive statistics for the different exposure models considered. The mean PM_2.5_ estimates are very similar across models. The RS models show the highest variation, as measured by the standard deviation and the interquartile range (IQR). The BMELUR model, however, has the largest overall range.

**Table 2:** Descriptive statistics for the exposure models after assignment to ACS CPS II participants.

Air pollution	*n*	Mean (SD)	Minimum	10th percentile	1st quartile	2nd quartile	3rd quartile	90th percentile	Maximum	IQR	Range
PM_2.5_ HBMCMAQ 02-04	668,629	12.1 (2.6)	2.8	8.7	10.4	12.1	14.0	15.2	21.4	3.6	18.6
PM_2.5_ BME 02-04	668,629	12.1 (2.6)	3.4	8.4	10.3	12.2	13.9	15.0	21.6	3.7	18.2
PM_2.5_ BMELUR 02-04	668,629	12.0 (2.7)	1.5	8.6	10.1	12.0	13.7	15.1	26.6	3.6	25.1
PM_2.5_ BMELUR CT 02-04	668,629	11.7 (2.8)	1.0	8.2	9.8	11.7	13.5	14.9	26.2	3.7	25.2
PM_2.5_ BMELURRS 02-04	668,629	12.0 (2.8)	3.2	8.4	10.0	11.9	13.8	15.2	24.4	3.7	21.2
PM_2.5_ BMELURRS CT 02-04	668,629	11.8 (2.8)	2.8	8.1	9.8	11.8	13.6	15.1	24.4	3.8	21.6
PM_2.5_ RS GWR CT 02-04	668,629	12.2 (3.2)	1.3	7.9	9.9	12.7	14.6	16.0	25.4	4.7	24.1
PM_2.5_ RS no GWR CT 02‑04	668,629	11.4 (3.6)	0.7	6.1	8.6	12.1	14.2	15.7	22.5	5.6	21.8
PM_2.5_ BMELUR 01-06	668,629	12.1 (2.6)	1.4	8.7	10.2	12.1	13.9	15.2	25.8	3.8	24.4
PM_2.5_ RS 01-06	668,629	11.9 (3.8)	1.9	7.0	9.0	11.8	14.7	16.9	24.6	5.7	22.6
Notes: ACS CPSII, American Cancer Society Cancer Prevention Study II; IQR, interquartile range.											

As shown in Table 3, moderately high to very high correlations exist among PM_2.5_ estimates from the five models (HBMCMAQ, BME, BMELUR, BMELURRS, and PM_2.5_ RS GWR CT 02-04) that included ground-based data in various ways, with correlations ranging from *r* = 0.71 to *r* = 0.94. The RS estimates without ground data show similarly high correlations with one another (*r* = 0.84). The two PM_2.5_ model groupings (ground-based vs. RS), however, have lower correlations with one another, ranging from *r* = 0.54 to 0.67. The one exception is a moderately high correlation between the two 1-km resolution RS surfaces (PM_2.5_ RS GWR CT 02-04 and PM_2.5_ RS no GWR CT 02-04), which exhibit a moderately high correlation of *r* = 0.78. Models that were assigned at both the participant residence and the CT level had very high correlations (*r* = 0.94 to 0.99).

**Table 3:** Correlations among the estimates of PM_2.5_ after assignment to ACS CPS II participants.

Air pollution	PM_2.5_ HBMCMAQ 02-04	PM_2.5_ BME 02-04	PM_2.5_ BMELUR 02-04	PM_2.5_ BMELUR CT 02-04	PM_2.5_ BMELURRS 02-04	PM_2.5_ BMELURRS CT 02-04	PM_2.5_ RS GWR CT 02‑04	PM_2.5_ RS no GWR CT 02‑04	PM_2.5_ BMELUR 01-06	PM_2.5_ RS 01-06
PM_2.5_ HBMCMAQ 02-04	1.00	0.88	0.84	0.82	0.85	0.85	0.71	0.59	0.84	0.63
PM_2.5_ BME 02-04		1.00	0.92	0.90	0.93	0.93	0.73	0.62	0.92	0.64
PM_2.5_ BMELUR 02-04			1.00	0.94	0.94	0.93	0.71	0.55	0.99	0.60
PM_2.5_ BMELUR CT 02-04				1.00	0.91	0.93	0.72	0.54	0.94	0.60
PM_2.5_ BMELURRS 02-04					1.00	0.99	0.72	0.58	0.93	0.66
PM_2.5_ BMELURRS CT 022‑04						1.00	0.73	0.58	0.92	0.67
PM_2.5_ RS GWR CT 02-04							1.00	0.78	0.74	0.72
PM_2.5_ RS no GWR CT 02‑04								1.00	0.59	0.84
PM_2.5_ BMELUR 01-06									1.00	0.62
PM_2.5_ RS 01-06										1.00
ACS CPSII, American Cancer Society Cancer Prevention Study II.										

Table 4 shows the results from the Cox regression modeling for mortality from diseases of the circulatory system and for IHD. We observed significant associations between particulate matter exposure and death in every model, although substantial variation exists among the magnitudes of the risk estimates. We found minor changes in the estimates when the ecologic confounders were added to the model, and given prior knowledge of how ecologic variables can affect mortality–air pollution associations, we report these as our primary results. For circulatory mortality, we observed the highest relative risks from the BMELUR [RR = 1.14, 95% confidence interval (CI): 1.11, 1.17], whereas the lowest relative risks resulted from the PM_2.5_ RS no GWR CT 02-04 estimate that excluded ground data (RR = 1.02, 95% CI: 1.00, 1.04). Relative risks from the other models are closer to those of the BMELUR (RR ~ 1.08–1.12). The ensemble estimate is the same as that of the BMELUR for circulatory mortality (RR = 1.14, 95% CI: 1.11, 1.17). As a sensitivity analysis, we also temporally matched the LURBME to the RS estimate from the 2001–2006 period. The results show slightly larger differences between the two estimates, but they are similar in magnitude to those obtained when the temporal periods differed (Table 4).

**Table 4:** Results of the Cox proportional hazard modeling with adjustment for individual or individual plus year 1990 ecologic covariates.

Air pollution	Diseases of the circulatory system *n* = 100,102				Ischemic heart disease *n* = 45,624			
	Fully-adjusted HR (95% CI)	AIC (1,587,000s)	Fully adjusted HR (95% CI) + 1990 ecological confounders	AIC (1,587,000s)	Fully-adjusted HR (95% CI)	AIC (726,000s)	Fully adjusted HR (95% CI) + 1990 ecological confounders	AIC (726,000s)
PM_2.5_ HBMCMAQ 02-04	1.09 (1.07, 1.12)	434	1.09 (1.06, 1.12)	094	1.15 (1.11, 1.19)	688	1.11 (1.07, 1.16)	315
PM_2.5_ BME 02-04	1.13 (1.10, 1.15)	388	1.12 (1.09, 1.15)	065	1.19 (1.15, 1.23)	650	1.15 (1.10, 1.19)	296
PM_2.5_ BMELUR 02-04	1.15 (1.13, 1.18)	340	1.14 (1.11, 1.17)	033	1.20 (1.16, 1.24)	636	1.15 (1.11, 1.19)	290
PM_2.5_ BMELUR CT 02-04	1.13 (1.11, 1.16)	364	1.12 (1.09, 1.15)	051	1.19 (1.15, 1.23)	643	1.14 (1.10, 1.18)	292
PM_2.5_ BMELURRS 02-04	1.12 (1.09, 1.14)	388	1.11 (1.08, 1.14)	066	1.18 (1.14, 1.22)	652	1.13 (1.09, 1.17)	297
PM_2.5_ BMELURRS CT 022‑04	1.11 (1.09, 1.13)	396	1.11 (1.08, 1.13)	068	1.17 (1.13, 1.20)	660	1.12 (1.08, 1.16)	301
PM_2.5_ RS GWR CT 02-04	1.09 (1.07, 1.11)	411	1.08 (1.06, 1.11)	088	1.10 (1.06, 1.13)	711	1.08 (1.05, 1.12)	321
PM_2.5_ RS no GWR CT 02‑04	1.04 (1.03, 1.06)	462	1.02 (1.00, 1.04)	131	1.09 (1.06, 1.12)	707	1.06 (1.02, 1.09)	331
PM_2.5_ BMELUR 01-06	1.16 (1.13, 1.19)	336	1.14 (1.11, 1.17)	036	1.20 (1.16, 1.25)	639	1.15 (1.11, 1.19)	293
PM_2.5_ RS 01-06	1.05 (1.04, 1.07)	447	1.05 (1.03, 1.07)	115	1.12 (1.10, 1.15)	658	1.10 (1.07, 1.14)	298
Ensemble estimate	1.16 (1.13, 1.19)	NA	1.14 (1.11, 1.17)	NA	1.20 (1.16, 1.24)	NA	1.15 (1.11, 1.19)	NA
Notes: ACS CPSII, American Cancer Society Cancer Prevention Study II; AIC, Akaike Information Criterion; CI, confidence interval; HR, hazard ratio. Hazard ratios expressed over a 10 μg/m^3^ increment. There are 43 variables in the model including PM_2.5_ for individual only and 55 in fully adjusted.								

Inclusion of the ecological variables had a relatively larger effect on the IHD estimate than on the circulatory mortality estimate. Although the inclusion of the ecological variables diminished the differences in the RRs between the exposure models, the pattern is similar, with PM_2.5_ RS no GWR CT 02-04 RS producing the lowest RRs. The differences between the other estimates, however, were somewhat smaller when the ecological covariates were included, with the BME kriging model and the BMELUR having the largest risks, followed closely by BMELURRS and the HBMCMAQ.

We also compared results across the inter-decile range (IDR) of exposure (see Table S2), which shows smaller differences between the estimates. We included this analysis to compare the models across the same range of exposure within their own distribution. The relative ordering is maintained for circulatory deaths, with the BMELUR and RS without ground monitors producing the largest and smallest RR estimates, respectively. For IHD deaths, after inclusion of the ecological covariates, many of the estimates are very similar, and the RS 01-06 model actually produced slightly larger relative risks (i.e., 1.1 versus 1.09), although the BMELUR model still had the lowest AIC out of all the models, indicating that this model was the best model fit.

We also restricted the analysis to only those participants who resided in cities with government monitoring stations (see Table S3 for descriptive statistics and Table 5 for results). This allowed us to compare the seven exposure models with those using only the spatial average per county, similar to earlier reports from the cohort that used only the central monitoring data (Jerrett et al. 2009; Pope et al. 2002). Here, we observed similar ordering; however, the RS model estimates tended to be even lower than before for this subset of the cohort. For circulatory deaths, the PM_2.5_ RS GWR CT 02-04 estimate was smaller than the county-wide average of the government monitor exposure estimates, which effectively produce only one estimate per county. For IHD, all RS estimates were smaller than the county-wide estimate.

**Table 5:** Cox proportional hazard model results restricted to those participants residing in a metropolitan area with a central monitor measurement of pollution.

Air pollution	Diseases of the circulatory system		Ischemic heart disease	
	Fully adjusted HR (95% CI) + 1990 ecological confounders	AIC (801,000s)	Fully adjusted HR (95% CI) + 1990 ecological confounders	AIC (373,000s)
Central monitor 99-00	1.08 (1.05, 1.12)	533	1.11 (1.06, 1.16)	292
PM_2.5_ HBMCMAQ 02-04	1.09 (1.05, 1.14)	536	1.09 (1.03, 1.16)	301
PM_2.5_ BME 02-04	1.11 (1.07, 1.15)	526	1.12 (1.06, 1.18)	294
PM_2.5_ BMELUR 02-04	1.12 (1.09, 1.16)	510	1.12 (1.06, 1.18)	291
PM_2.5_ BMELUR CT 02-04	1.12 (1.08, 1.16)	512	1.12 (1.07, 1.18)	289
PM_2.5_ BMELURRS 02-04	1.10 (1.06, 1.13)	527	1.11 (1.06, 1.16)	293
PM_2.5_ BMELURRS CT 022‑04	1.09 (1.06, 1.13)	527	1.10 (1.05, 1.15)	295
PM_2.5_ RS GWR CT 02-04	1.08 (1.04, 1.12)	539	1.09 (1.04, 1.15)	300
PM_2.5_ RS no GWR CT 02‑04	1.00 (0.97, 1.03)	555	1.03 (0.98, 1.07)	309
PM_2.5_ BMELUR 01-06	1.13 (1.09, 1.07)	511	1.12 (1.07, 1.18)	291
PM_2.5_ RS 01-06	1.03 (1.00, 1.06)	552	1.10 (1.06, 1.15)	288
Notes: AIC, Akaike Information Criterion; CI, confidence interval; HR, hazard ratio. Total *n* = 379,618 with 54,689 deaths from circulatory disease and 25,393 from ischemic heart disease. Hazard ratios expressed over a 10 μg/m^3^ increment. There are 43 variables in the model including PM_2.5_ for individual only and 55 in fully adjusted.				

The results indicate that models with a lower AIC (i.e., better model fit) generally had a higher RR estimate. For example, the increase in the RRs of circulatory and IHD deaths with respect to max(AIC) – AIC is clearly seen in Figure S1 (*R* ^2^ = 0.94). The AIC attained its maximum value (corresponding to the worst-fitting model) when the RS-based exposure assessment methods were used. The AIC had the lowest levels for the model including ground-based exposure methods, indicating improved model fit. Of these, the HBMCMAQ method had a larger AIC (poorer fit) than any of the BME methods, with BMELUR resulting in the smallest AIC (best overall fit) out of all the methods. In instances where we used geocoding to the residential address or to the CT, when we estimated exposures with both models of exposure assignment, we saw slight attenuation of the effects for the CT exposure assignment compared with the residential address. For example, with the BMELUR 02-04, the RR was 1.14 with the residential address, whereas with the CT assignment, it was 1.12. The higher AIC for the CT assignment indicates some degradation in model fit from the CT assignment.

As a sensitivity analysis, we included metropolitan area size as a covariate, given earlier findings suggesting that larger cities were associated with both lower mortality and higher pollution. We also included an elevation variable in this analysis. Inclusion of both variables separately or together had little impact on the size or overall pattern of the risks (see Tables S4 and S5 for details). For the two models with the highest and lowest RR estimates (the BMELUR and the RS models, respectively), we included a random effect at the county level. With the random effect, we observed even larger differences in the size of the RR between the BMELUR and RS exposure models than with the standard Cox model. As a final sensitivity analysis, we ran the models using ecological confounders from 2000 instead of from 1990 (see Table S6). The 2000 ecological variables exerted a slightly larger confounding effect on the PM_2.5_ relative risks in all models, but all results remained significantly elevated, and the ordering of the RRs between models was consistent with what we observed in the earlier analyses using ecological covariates from 1990.

Table S7 shows the results for the diabetes deaths. Here, without the ecological covariates, only risks from the LURBME models were significantly elevated, and all others included unity in the 95% confidence interval. With the addition of ecological covariates, many of the models did have significantly elevated risks, and the rank ordering among the models followed a similar pattern to that which we observed with circulatory and IHD deaths. In particular, the largest risks were observed in models using the BMELUR, with RR = 1.18 (95% CI: 1.05, 1.33), whereas the smallest were in models using RS with no ground data (RS no GWR CT 02-04), with RR = 1.01 (95% CI: 0.92, 1.11).

The concentration–response (C-R) curves for the BMELUR and RS models are shown in Figures S2 and S3. We investigated these curves to gain insights into the likely shape of the C-R curves. These curves were based on natural splines with 2 degrees of freedom. As expected from the model results, the BMELUR C-R curve has a steeper slope consistent with the larger coefficient versus the RS effect estimate. The RS model has a declining slope at approximately 15 μg/m^3^.

## Discussion

We found statistically significant positive associations between PM_2.5_ exposures and risk of death from circulatory disease and IHD with every exposure model tested for circulatory and IHD deaths. With the smaller number of diabetic deaths, we did not observe significant effects in all models for this outcome, but there were significant effects in many of the models after controlling for ecological confounding. These findings are in accord with those of several studies on this cohort, some of which used government monitors (Jerrett et al. 2009; Krewski et al. 2009; Pope et al. 2002), interpolation models (Jerrett et al. 2005), or hybrid land-use regressions that included ground-based information with traffic (Turner et al. 2014, 2016; Pope et al. 2015) and RS with land use (Jerrett et al. 2013). Our current findings strengthen the evidence base for a nonspurious association between PM_2.5_ exposure and mortality because estimates were significant for most models regardless of the exposure assessment method.

In general, the findings are in accord with existing evidence on the associations of PM_2.5_ with CVD outcomes, although the estimated associations here are somewhat larger. For example, the ensemble estimate for circulatory deaths was RR = 1.14 (95% CI: 1.11, 1.17), or a 14% increase, whereas a recent meta-analysis estimated a 10.6% (95% CI: 5.4, 16.0%) per the same exposure contrast of 10 μg/m^3^ (Hoek et al. 2013). Similarly, a recent analysis of another large, nationwide U.S. cohort found a 10% increase in CVD mortality with a RR = 1.10, 95% CI: 1.05, 1.15 (Thurston et al 2016). Results for RS models without ground monitors were approximately one-fifth to one-half the size of the meta-analysis estimates. Consistent with the Hoek et al. (2013) meta-analysis, we also observed slightly larger associations for IHD than for the broader circulatory category, although these estimates tended to be attenuated relatively more by the addition of ecological covariates than were those in the circulatory mortality category.

Although the findings of significant associations between PM_2.5_ and mortality appear consistent for most of the specific exposure models that were tested, the RR estimates varied markedly among the models. Compared with past studies, RRs here were larger for ground-based and more sophisticated hybrid models than for central monitors or RS exposure models alone. Among the ground-based exposure models tested, the HBMCMAQ model based on linear geostatistics had the poorest fit and the smallest effect size for circulatory and IHD deaths, whereas the BME models based on Bayesian epistemic knowledge blending had a better fit and a larger effect size. Of the BME exposure models, the best fit and largest association were obtained with BMELUR, a hybrid model containing information on traffic and local land use. On a per-microgram basis, the model containing traffic had associations that were > 2.5 times greater than the RS models for circulatory mortality. This stronger association might suggest a higher toxicity for the mixture of PM_2.5_ that originates from traffic or that fine-scale exposure estimates are needed to accurately assess health effects. We have used 10 μg/m^3^ as our primary comparison because this exposure increment shows the relative difference on a per-microgram basis. Even small increments to improve the overall accuracy of the exposure can be important for health effects assessment based on where these differences occur spatially. In the case of the BMELUR, the maximum contribution of the traffic variable to the overall model prediction was small, on the order of 1–1.5 μg/m^3^, but the spatial alignment of this to areas with dense traffic appears to capture potentially heightened toxicity from this source or the vulnerability of populations living in areas of high traffic or both, translating into much larger (2.5 times greater than RS) effects on a per-microgram basis. The differences between the models using the IDR were relatively smaller, which likely indicates that such comparisons were less able to determine essential differences between models that might have arisen from their ability to detect fine-scale variations near the source. RS models without ground data (i.e., RS no GWR CT 02-04 and RS 01-06) also had larger IDRs (i.e., 9.6 μg/m^3^ and 9.9 μg/m^3^, respectively), although the LURBME still had the largest range (26.6 μg/m^3^). Thus, the results also appear to be sensitive to the relative distributions of various exposure models. On a per-microgram basis, however, the relative rank ordering is clear and consistent.

Other emerging fine-resolution satellite retrievals (see, e.g., Lyapustin et al. 2011; Lee et al. 2016) may better resolve local aerosol sources, which might align the RS estimates more closely with ground-based models. RS is being increasingly combined with ground-based and LUR information for overall accuracy and to include finer resolution information (Beckerman et al. 2013b; Kloog et al. 2012b; Ma et al. 2014; van Donkelaar et al. 2015b). As these higher resolution models become more widely available, comparing these to other models such as the LURBME will be important for understanding whether, on a per-microgram basis, these hybrid models will detect health risks of similar magnitude to those predicted by other models. Our initial investigation here with the 1-km resolution RS GWR CT 02-04 model suggests that even with ground calibration, these models are not yet detecting risks of similar magnitude to those predicted by BMELUR, which included traffic data and smaller area prediction.

Our findings suggest caution against over-interpreting quantitative estimates of association between ambient PM_2.5_ and mortality based on a specific exposure assessment method. This caution is particularly necessary when estimating the air pollution–related burden of disease, which has relied on pooling concentration–response functions from studies with varying exposure assessment methods (Lim et al. 2012). Our work suggests that concentration–response modeling should be based on the most appropriate source of exposure information available. In regions where ground-based monitoring is sparse, the best available option may be to conduct an RS-based exposure assessment and use the best available concentration–response curve (Figure S2). Actual health risks, however, could be even larger than those derived from RS estimates without ground data. Hence, when there are sufficient ground-based monitoring data to calibrate an exposure model, hybrid exposure models should be used. Among these, we found that the HBMCMAQ exposure model had a poorer fit than the BME models; among the latter, the BMELUR model based on traffic and local land had the best fit and the largest effect size. In that case, our ground-based concentration–response curve (Figure S2) can therefore be considered to represent the best predictor of mortality. This conclusion is supported by the ensemble estimate that, owing to the superior fit of the BMELUR, ascribed nearly all the weight to this estimate of exposure. Because it reflects a larger effect size, that curve will attribute more deaths to PM_2.5_ than other models, particularly those based on RS with no ground data. Looking forward, an emerging global PM_2.5_ network (SPARTAN) is taking ground-based measurements to address key sources of uncertainty in RS estimates (Snider et al. 2015).

Our evaluation of the concentration–response functions for the BMELUR and RS models represents just two of several possible exposure–response functions and is intended purely to aid in visualizing whether, with identical spline functions, we observe different shapes of the exposure–response relationship between air pollution and mortality. We caution, however, against using these visual plots for understanding the underlying exposure–response functions. Such analyses would have to examine some weighted combination of several possible models rather than the single realization discussed here.

The present study has several strengths. We used a large data set with a long follow-up period and excellent control for covariates that could confound the air pollution–mortality relationship to estimate mortality associated with particulate air pollution. We also employed a comprehensive suite of exposure models ranging from those with no ground information, such as RS, to chemical transport models fused to ground data with Bayesian methods, to geostatistical kriging models, and finally to hybrid models that included either ground data only combined with advanced interpolation methods or some fusion of RS and land-use data. This suite of models covered most of the currently available exposure assessment methods likely to be employed in epidemiological analyses of mortality associated with long-term exposure to ambient air pollution.

The study also has several limitations. First, most exposure models were assigned at or near the end of the follow-up period, largely because of the lack of PM_2.5_ data before 1999. Previous analyses have shown that the relative spatial pattern likely remains constant in rank ordering over time (Jerrett et al. 2005). Relative declines in PM_2.5_ may have occurred unevenly across the country, potentially resulting in spatial heterogeneity that was not captured by the exposure models and, therefore, in differential levels of exposure error in each model. Moreover, study participants may have moved to higher- or lower-exposure areas, which could again impart error to the risk estimates. The extent to which either of these possible sources of error would influence the effect estimates from any of the models is difficult to determine. Some models had inherently larger grid areas for exposure assignment than others. Potential error sources in the RS GWR estimate include the exclusion of ground-based observations on spatial scales within 100 km and scaling to the 2002–2004 period. It would be instructive to revisit these limitations in future work. Similarly, one RS estimate, although contemporaneous with the ground estimates, was of longer duration and ran past the end of the follow-up period, and this may have introduced additional error that affected the relative size of the estimates. We did, however, conduct a sensitivity analysis in which we matched the LURBME to the exact temporal periods of the RS 01-06. The results from this sensitivity analysis were slightly stronger in terms of the difference between the two estimates but were essentially the same as those presented in the main results, suggesting that the temporal misalignment had a negligible impact on the overall patterning of the risks. We were unable to quantitatively assess the impact of measurement error with formal models owing to the lack of an externally valid “gold standard” to implement a regression calibration model (cf. Molitor et al. 2007).

## Conclusions

We found significant associations between PM_2.5_ exposure estimated using different models and risk of mortality. Relative risks estimated from exposure models using ground-based information were larger than those estimated with only RS.

The range of relative risks observed in this study also suggests new avenues for understanding the health effects of air pollution. This approach would follow the lead of climate models, whereby the various relative risk estimates could be combined or pooled into one estimate that would capture the range and uncertainty in the estimates. Similar approaches have been used to combine and assemble various estimates of future climate [Intergovernmental Panel on Climate Change (IPCC) 2014], where inherent uncertainty exists and no estimate is objectively superior. As an initial approach, we have developed ensemble estimates. Such approaches could be expanded to supply more accurate estimates of the effects of air pollution exposure on mortality with appropriate characterizations of model uncertainty.

## Supplemental Material

(444 KB) PDF
