# Supplementary material for: Comparing the Health Effects of Ambient Particulate Matter Estimated Using Ground-Based versus Remote Sensing Exposure Estimates
Source: Environ Health Perspect. 2016 Sep 9;125(4):552–9. doi: 10.1289/EHP575 (PMC5382001; doi:10.1289/EHP575)

Environ Health Perspect
DOI: 10.1289/EHP575

Note to readers with disabilities: *EHP* strives to ensure that all journal content is accessible to all readers. However, some figures and Supplemental Material published in *EHP* articles may not conform to 508 standards due to the complexity of the information being presented. If you need assistance accessing journal content, please contact ehp508@niehs.nih.gov. Our staff will work with you to assess and meet your accessibility needs within 3 working days.

Supplemental Material

Comparing the Health Effects of Ambient Particulate Matter Estimated Using Ground-Based versus Remote Sensing Exposure Estimates

Michael Jerrett, Michelle C. Turner, Bernardo S. Beckerman, C. Arden Pope III, Aaron van Donkelaar, Randall V. Martin, Marc Serre, Dan Crouse, Susan M. Gapstur, Daniel Krewski, W. Ryan Diver, Patricia F. Coogan, George D. Thurston, and Richard T. Burnett

Table of Contents

Methods. Detailed Description of the Individual and Ecological Variables included in the Cox proportional Hazards Models

Table S1. Distribution (n, %) of selected participant characteristics at enrollment (1982), ACS CPS II cohort, U.S., compared to those excluded from the analysis with commentary on the differences

Table S2. Results of the Cox Proportional Hazard Modeling with adjustment for individual or individual plus year 1990 ecologic covariates. Hazard ratios expressed over an IDR increment.

Table S3. Descriptive Statistics for the Central Monitor Exposure Estimates.

Table S4. Results of the Cox Proportional Hazard Modeling with adjustment for individual plus year 1990 ecologic covariates plus MSA size or elevation or RE model. Hazard ratios expressed over a 10 μg/m3 increment for diseases of the circulatory system.

Table S5. Results of the Cox Proportional Hazard Modeling with adjustment for

individual plus year 1990 ecologic covariates plus MSA size or elevation or RE model. Hazard ratios expressed over a 10 μg/m3 increment for ischemic heart disease.

Table S6. Results of the Cox Proportional Hazard Modeling with adjustment for individual or individual plus year 2000 ecologic covariates. Hazard ratios expressed over a 10 μg/m3 increment.

Table S7. Results of the Cox proportional hazard modeling with adjustment for individual or individual plus year 1990 ecologic covariates for mortality from diabetes. Hazard ratios expressed over a 10 $\mu g/m^3$ increment.

Figure S1. Fully-Adjusted hazard ratios (HR) from Table 2 for deaths of the circulatory system and IHD with HR plotted against AIC statistics to illustrate the effect of model fit on the HR among seven exposure models.

Figure S2. Concentration response plot for PM2.5 BMELUR 02-04 with two degrees of freedom for circulatory mortality

Figure S3. Concentration response plot for PM2.5 RS no GWR CT 02-04 with two degrees of freedom for circulatory mortality

Methods. Detailed Description of the Individual and Ecological Variables included in the Cox proportional Hazards Models

Every model included 13 terms to characterize current and former smoking status as well as smoking duration, amount, and age started smoking; a continuous variable was used to assess exposure to second hand cigarette smoke (hours/day exposed); a variable with seven terms was used to quantify exposure to $PM_{2.5}$ in the workplace for each of the subject's major lifetime occupation; another variable had self-reported exposure to dust and fumes at work; a variable with two terms representing marital status (separated/divorced/widowed or single versus married); a variable with two terms characterizing level of education (high school, more than high school versus less than high school); two body mass index (BMI) variables (linear and squared terms for BMI); a variable with two terms each assessing beer/wine/liquor consumption (beer, missing beer, wine, missing wine, liquor, missing liquor); and variables that indicate quintile ranges of a dietary vegetable/fruit/fiber index (four terms) and quintile ranges of dietary fat index (four terms) and one term for missing nutrition information.

These ecologic characteristics are documented elsewhere and included: median household income; percentage of people with < 125% of poverty-level income; percentage of persons over the age of 16 years who are unemployed; percentage of adults with less than 12th grade education; and percentage of the population who were Black or Hispanic. These ecological variables were entered at the ZIP code level and the ZIP code minus the county-level mean. The primary analysis used data from 1990 (see Table 4 in the main text). Sensitivity analysis using ecological covariates from 2000 is presented in Table S6.

Table S1. Distribution (n, %) of selected participant characteristics at enrollment (1982), ACS CPS II cohort, U.S., compared to those excluded

Characteristics	Included Participants (668,629) n (%)	Mean (SD) PM2.5 BMELUR 02-04	Excluded Participants (515,958) n (%)
Age (years)			
<40	29,602 (4.4)	12.2 (2.7)	24,738 (4.8)
40-49	137,509 (20.6)	11.9 (2.6)	101,427 (19.7)
50-59	245,008 (36.6)	12.0 (2.6)	174,066 (33.7)
60-69	117,988 (26.6)	12.0 (2.7)	139,216 (27.0)
70-79	66,498 (10.0)	12.0 (2.7)	62,807 (12.2)
≥80	12,024 (1.8)	12.1 (2.7)	13,704 (2.7)
Race			
White	632,508 (94.6)	11.9 (2.6)	472,077 (92.5)
Black	25,504 (3.8)	13.1 (2.4)	26,479 (5.2)
Other	10,617 (1.6)	12.3 (4.0)	11,735 (2.3)
Sex			
Male	292,580 (43.8)	11.9 (2.7)	215,738 (41.8)
Female	376,049 (56.2)	12.0 (2.7)	300,220 (58.2)
Education			
<High School	78,363 (11.7)	12.2 (2.6)	95,213 (19.1)
High School	207,579 (31.0)	12.0 (2.6)	168,846 (33.9)
≥High School	382,687 (57.2)	11.9 (2.7)	233,323 (46.9)
BMI (kg/m^2)			
<18.5	11,897 (1.8)	12.0 (2.7)	38,507 (7.5)
18.5-24.9	338,290 (50.6)	11.9 (2.7)	236,673 (45.9)
25-29.9	242,007 (36.2)	12.0 (2.6)	176,638 (34.2)
≥30	76,435 (11.4)	12.2 (2.6)	64,140 (12.4)
Marital Status			
Single	21,963 (3.3)	12.4 (2.6)	16,813 (3.3)
Married	563,805 (84.3)	11.9 (2.7)	419,205 (82.5)
Other	82,861 (12.4)	12.2 (2.7)	72,240 (14.2)
Cigarette Smoking Status			
Never	299,354 (44.8)	12.0 (2.7)	183,110 (35.5)
Current	129,778 (19.4)	12.1 (2.7)	110,299 (21.4)
Former	172,591 (25.8)	11.9 (2.7)	116,789 (22.6)
Pipe/cigar only	66,906 (10.0)	11.9 (2.6)	34,669 (6.7)

Note: The sum for excluded participants does not always equal the total due to missing data for some variables.

Commentary on Differences between Included and Excluded Subjects

In comparing those excluded to those in the analytical cohort, we see a higher proportion over 70 years in excluded. There is a slightly lower proportion of whites and higher proportion of blacks excluded. Slightly more of the excluded subjects are obese, but also more are underweight. There is also a lower proportion of never smokers excluded.

Table S2. Results of the Cox Proportional Hazard Modeling with adjustment for individual or individual plus year 1990 ecologic covariates. Hazard ratios expressed over an IDR increment.

	Diseases of the circulatory system				Ischemic heart disease			
	N=100,102				N=45,624			
Air Pollution	Fully-adjusted HR (95% CI)	AIC (1,587,000s)	Fully-adjusted HR (95% CI) + 1990 Ecological Confounders	AIC (1,587,000s)	Fully-adjusted HR (95% CI)	AIC (726,000s)	Fully-adjusted HR (95% CI) + 1990 Ecological Confounders	AIC (726,000s)
PM2.5 HBMCMAQ 02-04	1.06 (1.04-1.07)	434	1.06 (1.04-1.08)	94	1.09 (1.07-1.12)	688	1.07 (1.04-1.10)	315
PM2.5 BME 02-04	1.08 (1.06-1.10)	388	1.08 (1.06-1.10)	65	1.12 (1.10-1.15)	650	1.09 (1.07-1.12)	296
PM2.5 BMELUR 02-04	1.10 (1.08-1.11)	340	1.09 (1.07-1.11)	33	1.13 (1.10-1.15)	636	1.09 (1.07-1.12)	290
PM2.5 BMELUR CT 02-04	1.09 (1.07-1.10)	364	1.08 (1.06-1.10)	51	1.12 (1.10-1.15)	643	1.09 (1.07-1.12)	292
PM2.5 BMELURRS 02-04	1.08 (1.06-1.10)	388	1.07 (1.06-1.09)	66	1.12 (1.09-1.14)	652	1.09 (1.06-1.12)	297
PM2.5 BMELURRS CT 02-04	1.08 (1.06-1.09)	396	1.07 (1.05-1.09)	68	1.11 (1.09-1.14)	660	1.09 (1.06-1.11)	301
PM2.5 RS GWR CT 02-04	1.07 (1.06-1.09)	411	1.07 (1.05-1.09)	88	1.08 (1.05-1.10)	711	1.07 (1.04-1.10)	321
PM2.5 RS no GWR CT 02-04	1.04 (1.03-1.06)	462	1.02 (1.00-1.04)	131	1.08 (1.06-1.11)	707	1.05 (1.02-1.09)	331
PM2.5 BMELUR 01-06	1.10 (1.08-1.12)	336	1.09 (1.07-1.11)	36	1.13 (1.10-1.15)	639	1.09 (1.07-1.12)	293
PM2.5 RS 01-06	1.05 (1.04-1.07)	447	1.05 (1.02-1.07)	115	1.12 (1.10-1.15)	658	1.10 (1.07-1.14)	298

Note 1: There are 43 variables in the model including $PM_{2.5}$ for individual only and 55 in fully adjusted.

Table S3. Descriptive Statistics for the Central Monitor Exposure Estimate.

Air Pollution	N	Mean (SD)	Minimum	10th percentile	1st quartile	2nd quartile	3rd quartile	90th percentile	Maximum	IQR	Range
Central monitor 99-00	379618	14.0 (3.0)	5.8	10.2	11.8	14.4	16.0	17.9	22.2	4.2	16.4

Table S4. Results of the Cox Proportional Hazard Modeling with adjustment for individual plus year 1990 ecologic covariates plus MSA size or elevation or RE model.

	Diseases of the circulatory system				
	N=100,102				
Air Pollution	Fully-adjusted HR (95% CI) + 1990 Ecologicals	+ MSA size indicator	+ elevation 90th percentile	+ elevation 95th percentile	Fully-adjusted HR (95% CI) + 1990 Ecological Confounders RE model (county)*
PM2.5 HBMCMAQ 02-04	1.09 (1.06-1.12)	1.08 (1.05-1.11)	1.09 (1.07-1.13)	1.10 (1.07-1.13)	-
PM2.5 BME 02-04	1.12 (1.09-1.15)	1.11 (1.08-1.14)	1.12 (1.09-1.15)	1.12 (1.09-1.15)	-
PM2.5 BMELUR 02-04	1.14 (1.11-1.17)	1.13 (1.10-1.16)	1.14 (1.11-1.17)	1.14 (1.11-1.17)	1.16 (1.12-1.20)<br>0.00518791
PM2.5 BMELUR CT 02-04	1.12 (1.09-1.15)	1.11 (1.09-1.14)	1.12 (1.10-1.15)	1.12 (1.10-1.15)	1.12 (1.09-1.16)<br>0.0051866
PM2.5 BMELUR 01-06	1.14 (1.11-1.17)	1.13 (1.10-1.17)	1.14 (1.11-1.17)	1.14 (1.11-1.17)	-
PM2.5 BMELURRS 02-04	1.11 (1.08-1.14)	1.10 (1.07-1.13)	1.11 (1.08-1.14)	1.11 (1.08-1.14)	-
PM2.5 BMELURRS CT 02-04	1.11 (1.08-1.13)	1.10 (1.07-1.13)	1.11 (1.08-1.13)	1.11 (1.08-1.13)	-
PM2.5 RS 01-06	1.05 (1.03-1.07)	1.04 (1.02-1.06)	1.05 (1.02-1.07)	1.05 (1.02-1.07)	-
PM2.5 RS GWR CT 02-04	1.08 (1.06-1.11)	1.08 (1.05-1.10)	1.08 (1.06-1.11)	1.08 (1.06-1.11)	1.08 (1.05-1.11)<br>0.00547513
PM2.5 RS no GWR CT 02-04	1.02 (1.00-1.04)	1.01 (0.99-1.03)	1.02 (1.00-1.04)	1.02 (1.00-1.04)	1.02 (0.99-1.05)<br>0.00585872

Note 1: adjustments are not cumulative. MSA size only, elevation only etc.
Note 2: Hazard ratios expressed over a 10 μg/m3 increment.
* Results shown only for the BMELUR estimates assigned to the CT and the geocoded residence and RS 1 km surfaces for time-matched comparison. Models shown in this column have the AIC below the HR for comparison of model fit between the models.

Table S5. Results of the Cox Proportional Hazard Modeling with adjustment for individual plus year 1990 ecologic covariates plus MSA size or elevation or RE model.

	Ischemic Heart Disease				
	N=45,624				
Air Pollution	Fully-adjusted HR (95% CI) + 1990 Ecologicals	+ MSA size indicator	+ elevation 90th percentile	+ elevation 95th percentile	Fully-adjusted HR (95% CI) + 1990 Ecological Confounders RE model (county)*
PM2.5 HBMCMAQ 02-04	1.11 (1.07-1.16)	1.08 (1.04-1.13)	1.12 (1.07-1.16)	1.12 (1.08-1.17)	-
PM2.5 BME 02-04	1.15 (1.10-1.19)	1.12 (1.08-1.17)	1.15 (1.10-1.19)	1.15 (1.11-1.19)	-
PM2.5 BMELUR 02-04	1.15 (1.11-1.19)	1.12 (1.08-1.17)	1.15 (1.11-1.20)	1.15 (1.11-1.20)	1.16 (1.10-1.22)<br>0.0141186
PM2.5 BMELUR CT 02-04	1.14 (1.10-1.18)	1.12 (1.08-1.16)	1.14 (1.10-1.19)	1.14 (1.10-1.19)	1.14 (1.09-1.20)<br>0.0141060
PM2.5 BMELUR 01-06	1.15 (1.11-1.19)	1.12 (1.08-1.17)	1.15 (1.11-1.20)	1.15 (1.11-1.20)	-
PM2.5 BMELURRS 02-04	1.13 (1.09-1.17)	1.11 (1.07-1.15)	1.14 (1.09-1.18)	1.14 (1.10-1.18)	-
PM2.5 BMELURRS CT 02-04	1.12 (1.08-1.16)	1.10 (1.06-1.14)	1.13 (1.09-1.17)	1.13 (1.09-1.17)	-
PM2.5 RS 01-06	1.10 (1.07-1.14)	1.08 (1.05-1.12)	1.10 (1.07-114)	1.10 (1.07-1.14)	-
PM2.5 RS GWR CT 02-04	1.08 (1.05-1.12)	1.06 (1.03-1.10)	1.09 (1.05-1.12)	1.09 (1.05-1.13)	1.08 (1.03-1.12)<br>0.0145285
PM2.5 RS no GWR CT 02-04	1.06 (1.02-1.09)	1.04 (1.00-1.07)	1.06 (1.02-1.09)	1.06 (1.02-1.09)	1.06 (1.01-1.10)<br>0.0144213

Note 1: Adjustments are not cumulative. MSA size only, elevation only etc.
Note 2: Hazard ratios expressed over a 10 μg/m^3 increment.
Results shown only for the BMELUR estimates assigned to the CT and the geocoded residence and RS 1 km surfaces for time-matched comparison. Models shown in this column have the AIC below the HR for comparison of model fit between the models.

Table S6. Results of the Cox Proportional Hazard Modeling with adjustment for individual or individual plus year 2000 ecologic covariates. Hazard ratios expressed over a 10 μg/m3 increment.

	Diseases of the circulatory system				Ischemic heart disease			
	N=100,102				N=45,624			
Air Pollution	Fully-adjusted HR (95% CI)	AIC (1,587,000s)	Fully-adjusted HR (95% CI) + 2000 Ecological Confounders	AIC (1,587,000s)	Fully-adjusted HR (95% CI)	AIC (726,000s)	Fully-adjusted HR (95% CI) + 2000 Ecological Confounders	AIC (726,000s)
PM2.5 HBMCMAQ 02-04	1.09 (1.07-1.12)	434	1.07 (1.04-1.10)	92	1.15 (1.11-1.19)	688	1.08 (1.04-1.13)	335
PM2.5 BME 02-04	1.13 (1.10-1.15)	388	1.10 (1.07-1.13)	69	1.19 (1.15-1.23)	650	1.12 (1.07-1.16)	320
PM2.5 BMELUR 02-04	1.15 (1.13-1.18)	340	1.12 (1.09-1.15)	40	1.20 (1.16-1.24)	636	1.13 (1.08-1.17)	314
PM2.5 BMELUR CT 02-04	1.13 (1.11-1.16)	364	1.11 (1.08-1.13)	55	1.19 (1.15-1.23)	643	1.12 (1.08-1.16)	314
PM2.5 BMELURRS 02-04	1.12 (1.09-1.14)	388	1.09 (1.07-1.12)	68	1.18 (1.14-1.22)	652	1.11 (1.07-1.15)	319
PM2.5 BMELURRS CT 02-04	1.11 (1.09-1.13)	396	1.09 (1.06-1.12)	70	1.17 (1.13-1.20)	660	1.10 (1.06-1.14)	322
PM2.5 RS GWR CT 02-04	1.09 (1.07-1.11)	411	1.07 (1.04-1.09)	86	1.10 (1.06-1.13)	711	1.06 (1.02-1.10)	339
PM2.5 RS no GWR CT 02-04	1.04 (1.03-1.06)	462	1.00 (0.98-1.02)	116	1.09 (1.06-1.12)	707	1.03 (1.00-1.06)	347
PM2.5 BMELUR 01-06	1.16 (1.13-1.19)	336	1.12 (1.09-1.15)	44	1.20 (1.16-1.25)	639	1.12 (1.08-1.17)	317
PM2.5 RS 01-06	1.05 (1.04-1.07)	447	1.03 (1.01-1.05)	107	1.12 (1.10-1.15)	658	1.09 (1.06-1.12)	319

Note there are 43 variables in the model including PM2.5 for individual only and 55 in fully adjusted.

Table S7. Results of the Cox proportional hazard modeling with adjustment for individual or individual plus year 1990 ecologic covariates for mortality from diabetes. Hazard ratios expressed over a 10 μg/m^3 increment.

	Diabetes (ICD XX)			
	N=4,886			
Air Pollution	Fully-adjusted HR (95% CI)	AIC (77,000s)	Fully-adjusted HR (95% CI) + 1990 Ecological Confounders	AIC (77,000s)
PM2.5 HBMCMAQ 02-04	1.04 (0.93-1.16)	604	1.15 (1.01-1.30)	578
PM2.5 BME 02-04	1.02 (0.91-1.13)	604	1.08 (0.96-1.22)	581
PM2.5 BMELUR 02-04	1.12 (1.01-1.24)	600	1.18 (1.05-1.33)	575
PM2.5 BMELUR CT 02-04	1.10 (1.00-1.22)	601	1.17 (1.05-1.30)	575
PM2.5 BMELURRS 02-04	1.06 (0.96-1.17)	603	1.12 (1.00-1.25)	579
PM2.5 BMELURRS CT 02-04	1.06 (0.96-1.17)	603	1.13 (1.01-1.25)	578
PM2.5 RS GWR CT 02-04	1.01 (0.92-1.10)	604	1.11 (1.00-1.24)	579
PM2.5 RS no GWR CT 02-04	0.97 (0.89-1.05)	604	1.01 (0.92-1.11)	583
PM2.5 BMELUR 01-06	1.11 (1.00-1.24)	601	1.18 (1.05-1.33)	575
PM2.5 RS 01-06	1.00 (0.93-1.08)	604	1.08 (0.98-1.18)	580

Note 1: There are 43 variables in the model including $PM_{2.5}$ for individual only and 55 in fully adjusted.

Figure S1. Fully-Adjusted hazard ratios (HR) from Table 2 for deaths of the circulatory system and IHD with HR plotted against AIC statistics to illustrate the effect of model fit on the HR among seven exposure models.

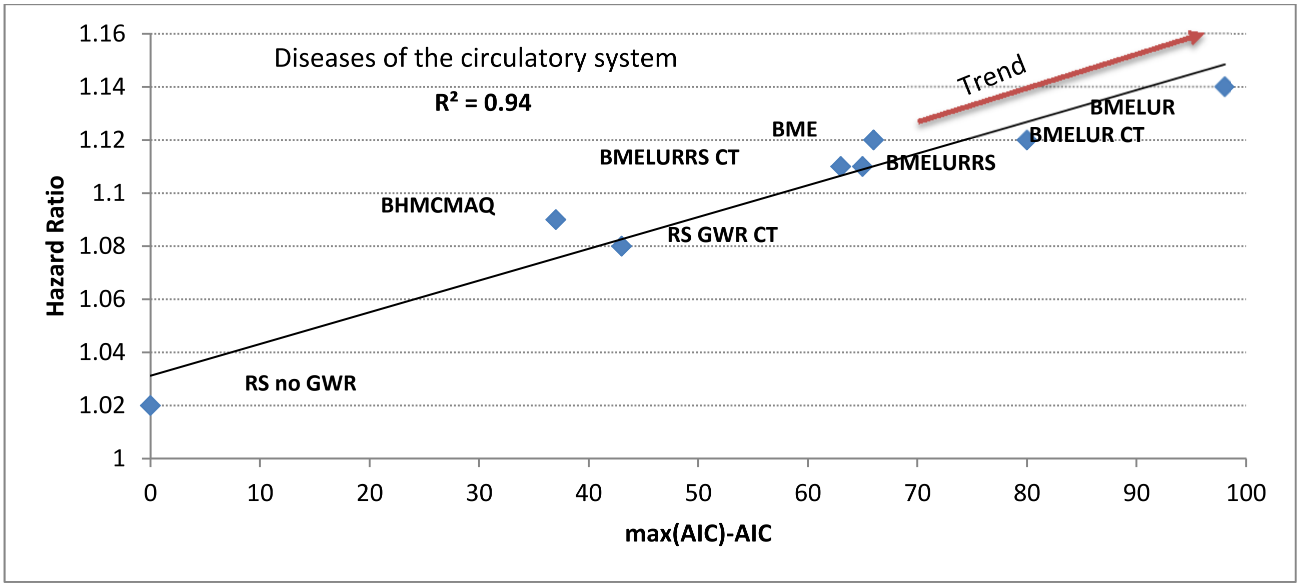

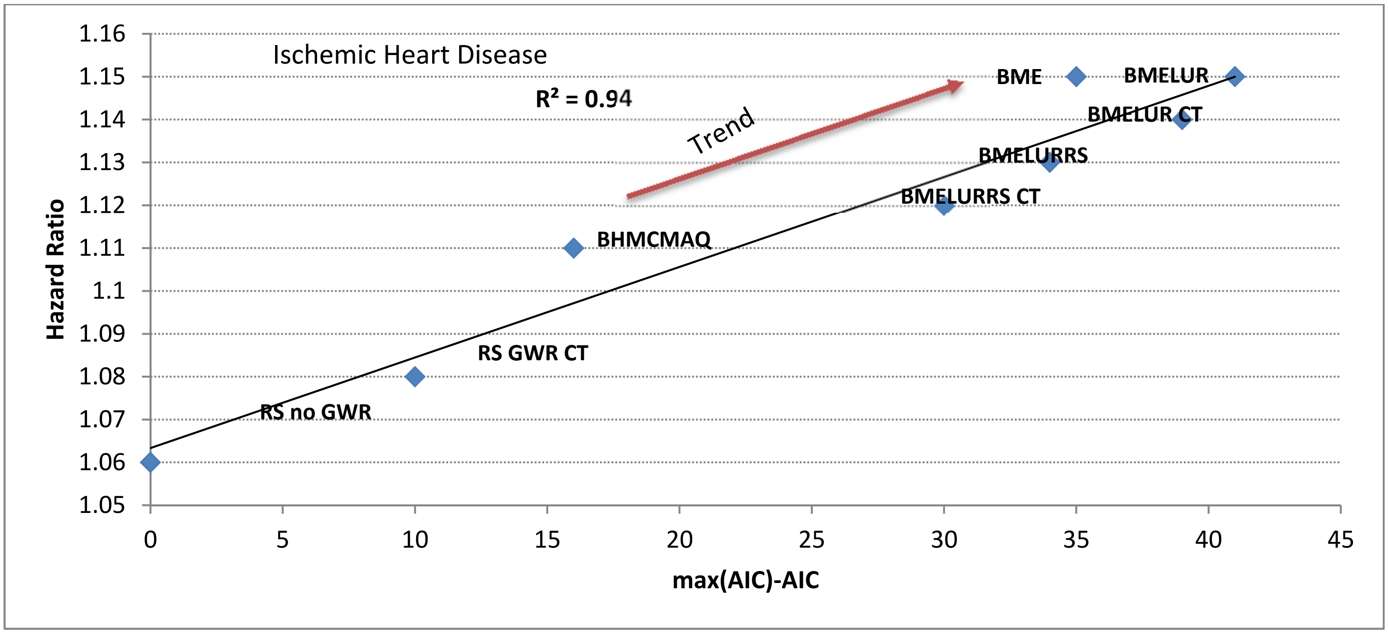

Figure S2. Concentration response plot for PM2.5 BMELUR 02-04 with two degrees of freedom for circulatory mortality

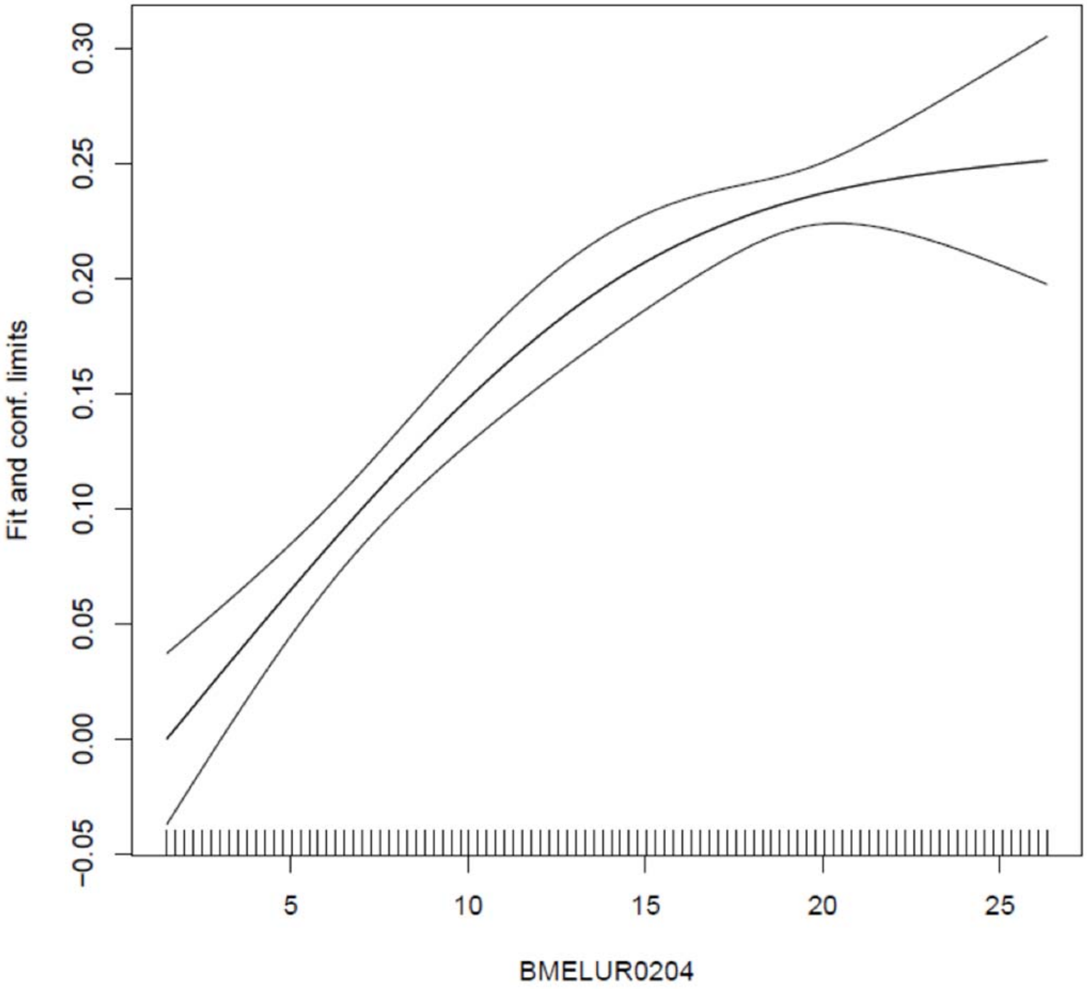

Figure S3. Concentration response plot for PM2.5 RS no GWR CT 02-04 with two degrees of freedom for circulatory mortality

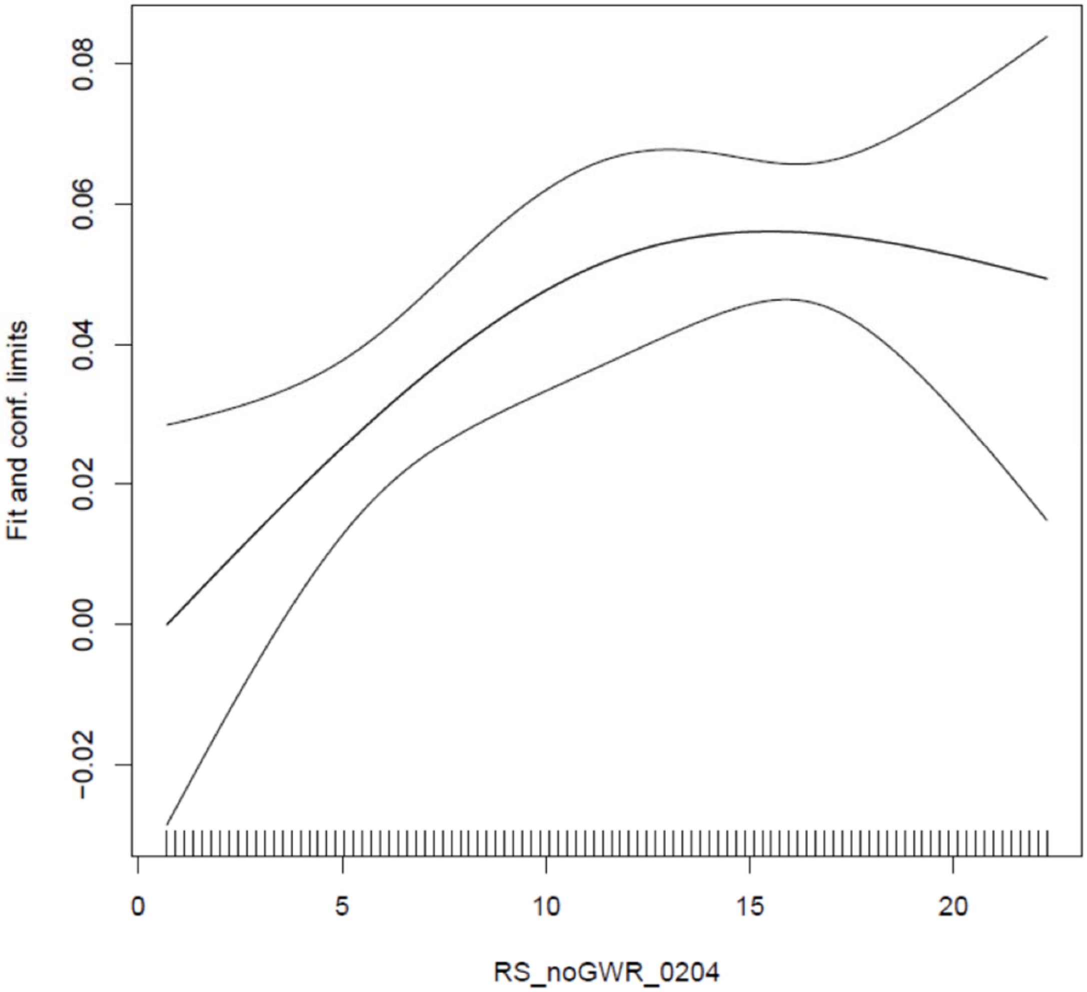

Supplement: (444 KB) PDF [file EHP575.s001.acco.pdf]
